# Changes in the growth and physiological property of tea tree after aviation mutagenesis and screening and functional verification of its characteristic hormones

**DOI:** 10.3389/fpls.2024.1402451

**Published:** 2024-07-24

**Authors:** Miao Jia, Yiling Chen, Qi Zhang, Yuhua Wang, Mingzhe Li, Xiaomin Pang, Lei Hong, Shaoxiong Lin, Xiaoli Jia, Jianghua Ye, Haibin Wang

**Affiliations:** ^1^ College of Tea and Food, Wuyi University, Wuyishan, China; ^2^ College of Life Science, Longyan University, Longyan, China; ^3^ College of JunCao Science and Ecology, Fujian Agriculture and Forestry University, Fuzhou, China

**Keywords:** *Camellia sinensis*, growth indexes, physiological indexes, characteristic hormones, aviation mutagenesis

## Abstract

Aerospace breeding is a breeding technique that utilizes a spacecraft to position plants in a space environment for mutagenesis, which is conducive to rapid mutagenesis for the screening of superior plant varieties. In this study, tea trees with aviation mutagenesis (TM) and those without aviation mutagenesis (CK) were selected as research subjects to analyze the effects of aviation mutagenesis on the growth, physiological properties, and hormone metabolism of tea trees, and to further screen the characteristic hormones and validate their functions. The results showed that the leaf length, leaf width, and leaf area of TM tea trees were significantly larger than those of CK. The growth indexes, the photosynthetic physiological indexes (i.e., chlorophyll content, intercellular CO_2_ concentration, stomatal conductance, transpiration rate, and photosynthetic rate), and the resistance physiological indexes (i.e., superoxide dismutase, peroxidase, catalase, and soluble sugar) were significantly higher in TM than in CK. Hormone metabolome analysis showed that four characteristic hormones distinguished CK from TM, namely, l-tryptophan, indole, salicylic acid, and salicylic acid 2-*O*-β-glucoside, all of which were significantly more abundant in TM than in CK. These four characteristic hormones were significantly and positively correlated with the growth indexes, tea yield, and the photosynthetic and resistance physiological indexes of tea trees. The leaf area, chlorophyll content, photosynthetic rate, and superoxide dismutase activity of tea tree seedlings after spraying with the four characteristic hormones were significantly increased, in which salicylic acid and salicylic acid 2-*O*-β-glucoside were more favorable to increase the leaf area and superoxide dismutase activity, while l-tryptophan and indole were more favorable to increase the leaf chlorophyll content and photosynthetic rate. It can be observed that aviation mutagenesis improves the accumulation of the characteristic hormones of tea trees, enhances their photosynthetic capacity, improves their resistance, promotes their growth, and then improves the tea yield.

## 1 Introduction

Aerospace breeding, also known as space technology breeding or space breeding, is a new crop breeding technology that utilizes the space environment that can be reached by a return spacecraft or a high-altitude balloon, among others, to mutagenize plants in order to produce mutations and then to further selectively breed the mutagenized plants on Earth to obtain new germplasm and new materials and to cultivate new varieties. Any mutation that occurs in the plant material in space is known as aviation mutagenesis (Mohanta et al., 2021). Plant germplasm resources are extremely important for the development of agriculture and are important materials for the cultivation of new plant varieties and for biological research. Moreover, aerospace mutation breeding is of great significance for enriching plant germplasm resources. During the process of aviation mutagenesis, plants are exposed to extreme conditions such as high levels of radiation, microgravity, high vacuum, and extreme fluctuations in temperature, which can highly affect their genes and physiological properties (Prasad et al., 2021). This change facilitates the rapid and efficient screening of high-quality germplasm resources (Fang and Zhou, 2004; Furukawa et al., 2020). Therefore, scientists around the world have long attached great importance to the use of aviation mutagenesis for the selection and breeding of certain plant varieties with special advantages (Xia et al., 2021). Furthermore, China has also made notable achievements in the field of plant aviation mutation breeding. To date, China has successfully developed over 200 new varieties with superior traits. These varieties have been widely demonstrated and promoted in a total area of over 2 million square hectometers, thereby significantly boosting the agricultural development of China (Ma et al., 2021; Mohanta et al., 2021). In 2003, China initiated the aviation mutation breeding of tea trees, resulting in the successful testing of the first batch of tea trees through the launch of “Shenzhou V.” During the period from 2003 to 2023, a total of 12 batches of different varieties of tea trees underwent aviation mutation breeding. Although this is an ambitious work, it is disappointing that relatively little scientific literature has been published in the last two decades to study the effects of aviation mutagenesis on the growth and physiological property of tea trees. It is conceivable that mutagenized tea trees may lack any significant characteristics or that the lack of research on this issue has led to confusion in the development of space breeding of tea trees. Therefore, the selection of superior tea tree varieties through space mutagenesis has become a formidable challenge.

Wuyi Mountain in Fujian Province is the birthplace of oolong tea and is an important tea-producing area in China. Previous studies have found that mutagenized tea trees have undergone significant changes in morphology and exhibited some excellent traits (Liu et al., 2020). Tea tree growth is closely related to photosynthetic capacity and resistance, and high photosynthetic capacity and resistance are more conducive to the promotion of tea tree growth (Zhang et al., 2023). In addition, hormones regulate the physiological mechanisms, photosynthetic capacity, and the related resistance of tea trees during their growth and development (Ahmad et al., 2022; Waadt et al., 2022; Jia et al., 2023). The authors hypothesized that advantages in the leaf morphology and growth trend of tea trees after aviation mutagenesis could contribute to higher tea tree yields and that these advantages may be created by differences in the synthesis and accumulation of hormones in tea trees, leading to changes in their physiological properties.

Accordingly, this study was conducted to measure the growth indexes, photosynthetic physiological indexes, resistance physiological indexes, and hormone metabolome of Dahongpao tea trees with aviation mutagenesis (TM) and without aviation mutagenesis (CK) to analyze and obtain the characteristic hormones that differentiate TM from CK, and to further perform correlation analysis. On this basis, exogenous spraying of Dahongpao tea tree seedlings without aviation mutagenesis using characteristic hormones was performed to measure the physiological indexes of tea trees and to analyze the effects of these characteristic hormones on the physiological properties of tea tree seedlings in order to verify previous results. This study will provide a reference for the development of aviation mutagenesis of tea trees and lay a foundation for the application and popularization of aviation mutagenesis of tea trees.

## 2 Materials and methods

### 2.1 Experimental site and sample sampling

This study was conducted on tea trees with (TM) and without (CK) aviation mutagenesis. The specific process of tea tree aerospace mutagenesis is as follows: at 5:58 p.m. on November 1, 2011, Dahongpao tea tree seeds were launched with the unmanned spacecraft “Shenzhou VIII”, and at 5:58 p.m. on November 3, 2011, “Shenzhou VIII” and the target aircraft “Tiangong I” had a space rendezvous and docking to form a combination operating for 12 days. At 6:30 p.m. on November 16, 2011, the combination was successfully separated and the return capsule was on the ground at 7:32 p.m. on November 17, 2011, with a total flight time of 16 days, 13 h, and 34 min. The experiment site was at the “Spaceflight Breeding Experimental Base of Xianmingyan Tea Factory” in Wuyishan City, Nanping, Fujian Province, China (117°59′ 47.7″ E, 27°44′8.4″ N). The tea tree variety used was Dahongpao (*Camellia sinensis*). In May 2023, the inverted first three leaves of TM and CK tea trees were collected. This was performed in order to observe the morphology of the tea tree leaves and to determine the growth and photosynthetic physiological indexes of the tea trees. In addition, the inverted second leaves of TM and CK tea trees were collected and immediately placed in liquid nitrogen to determine the physiological resistance indexes and the hormone metabolome of the tea trees.

### 2.2 Determination of tea tree growth indexes

The growth indexes of the tea trees were mainly determined based on the leaf number, the bud density, the hundred-bud weight, and the leaf area and yield. The specific methods referred to the “*Technical specification for tea production, processing and safety testing*” (Wang et al., 2020). A total of 20 mature new shoots were randomly selected and observed for the number of leaves. The average value, i.e., one replicate, of eight independent replicates was calculated. Bud density was calculated by randomly selecting an area of 20 m^2^ in the tea plantation, determining the number of buds in the area, and then converting to obtain the bud density, with eight independent replicates for each sample. The hundred-bud weight was determined by weighing 100 randomly selected buds, i.e., one replicate and six independent replicates for each sample. Leaf area was measured by randomly selecting the 10 inverted second leaves of tea trees, determining the leaf length and leaf width as leaf area = leaf length × leaf width × 0.7, and taking the mean value, i.e., one replicate and six independent replicates for each sample. Yield was calculated by randomly selecting 10 m^2^ of all areas with planted tea trees, picking and weighing according to the Wuyi Rock Tea picking standards, and then converting the tea yield, with six independent replicates for each sample.

### 2.3 Determination of the photosynthetic physiological indexes of tea tree leaves

The photosynthetic physiological indexes of the tea tree leaves were determined based on the chlorophyll content, intercellular CO_2_ concentration, stomatal conductance, transpiration rate, and photosynthetic rate, with six replicates for each sample. The chlorophyll content was determined using a chlorophyll analyzer (SPAD-502 PLUS, Tokyo, Japan). The other photosynthetic indexes were determined using the LI-6400XT Portable Photosynthesis System (Li-Cor, Lincoln, NE, USA). The parameters measured showed the following: ambient CO_2_ concentration, 360 ppm; photon flux density, 1,500 μmol/m^2^ s; and vapor pressure deficit (VPD) in the vessel, <1 kPa. Leaf temperature was kept at 25–26°C.

### 2.4 Determination of the physiological indexes of resistance in tea tree leaves

The physiological indexes of resistance in the tea leaves were determined for superoxide dismutase, catalase, peroxidase, soluble sugar, and malondialdehyde, with three replicates per sample. The superoxide dismutase, catalase, and peroxidase activities were determined using an enzyme-linked immunosorbent assay (ELISA) kit (Shanghai Preferred Biotechnology Co., Ltd., Shanghai, China). Briefly, 0.3 g of fresh tea tree leaves were weighed for each sample, extracted using different ELISA kits and assayed using a multifunctional enzyme labeling instrument (BioTek Synergy2 Gene 5, Winooski, VT, USA). Enzyme activity was expressed as units per gram. Superoxide dismutase, catalase, and peroxidase were measured at 560, 240, and 470 nm, respectively. The soluble sugar content was determined using anthrone colorimetry at a wavelength of 630 nm and then converted to its content. The malondialdehyde content was determined with the thiobarbituric acid method at wavelengths of 450, 532, and 600 nm and then converted to its content.

### 2.5 Hormone metabolome analysis of tea tree leaves

For the determination of the hormone metabolome of the tea tree leaves, 50 mg of ground leaves was weighed, 1 mL of a methanol/water/formic acid (15:4:1, *v*/*v*/*v*) extractant was added, vortexed and oscillated for 10 min, and then centrifuged at 12,000 rpm for 5 min. The supernatant was then fixed to 100 μL with 80% methanol, passed through a 0.22-μm filter membrane, and the hormone content determined using liquid chromatography/tandem mass spectrometry (LC-MS/MS) (Floková et al., 2014; Li et al., 2016). There were three independent replicates per sample.

The LC-MS/MS systems used were primarily ultra-performance liquid chromatography (ExionLC™ AD, AB Sciex, Concord, Canada) and tandem mass spectrometry (QTRAP® 6500+, AB Sciex, Concord, Canada). The column used for the liquid phase was a Waters ACQUITY UPLC HSS T3 C18 column (1.8 µm, 100 mm × 2.1 mm i.d.). The mobile phases were set up as phase A and phase B, which consisted of ultrapure water containing 0.04% acetic acid and acetonitrile containing 0.04% acetic acid, respectively. The ratio of phase A/phase B was set up as a volumetric ratio in the gradient elution process, and the gradient elution conditions were 95:5, 5:95, and 95:5 for A/B at 0, 8.0, and 9.1 min, respectively. The flow rate was set at 0.35 mL/min, the column temperature was 40°C, and the injection volume was 2 μL. For the mass spectrometry parameters (Šimura et al., 2018), the temperature for electrospray ionization was set to 550°C, the mass spectrometry voltages were set to 5,500 V (positive ion mode) and −4,500 V (negative ion mode), and the curtain gas was set to 35 psi. Scanning detection was performed for each ion according to the optimized declustering potential and collision energy.

The different hormone standards were configured with 10 gradient concentrations in the range of 0.01–500 ng/mL and were determined according to the LC-MS/MS method described above for sample detection. Quantitative signal chromatographic peak intensity data corresponding to each concentration of the standard were obtained to plot the standard curves of the different hormones (**Supplementary Table S1**). The integral peak areas detected in the samples were replaced with the standard curves to obtain the contents of the different hormones.

### 2.6 Exogenous spraying of tea seedlings with characteristic hormones

Based on the analysis of the experimental results, four characteristic hormones, i.e., l-tryptophan, indole, salicylic acid, and salicylic acid 2-*O*-β-glucoside, were obtained to distinguish between TM and CK trees. The contents of all four characteristic hormones in TM were significantly higher than those in CK. Therefore, based on the contents of l-tryptophan (17.36 mg/g), indole (3.49 mg/g), salicylic acid (3.25 mg/g), and salicylic acid 2-*O*-β-glucoside (8.46 mg/g) measured in the hormone metabolome of TM trees, the hormone solution was set at the appropriate concentration for the exogenous spraying of tea tree seedlings. The final concentrations of l-tryptophan, indole, salicylic acid, and salicylic acid 2-*O*-β-glucoside were 17.35, 3.50, 3.25, and 8.45 g/L, respectively.

Soils from the tea plantations that had not been planted with tea trees were collected, air-dried, ground, and sieved through a 40-mesh sieve. The soil organic matter, total nitrogen, total phosphorus, and total potassium contents were 8.43, 1.08, 0.62, and 1.91 g/kg, respectively, while the quick nitrogen, quick phosphorus, and quick potassium contents were 56.38, 11.42, and 89.35 mg/kg, respectively. The soil was packed into pots of 10 kg soil per pot, and 1-year-old, relatively uniform Dahongpao tea tree seedlings (not aviation-mutagenized) were selected and transplanted into pots with six plants each pot. After the transplanted tea seedlings resumed growth for 30 days, the tea seedlings were exogenously sprayed with different hormone solutions, with tea seedlings sprayed with distilled water used as the controls, with three independent replicates for each treatment. The hormone solutions were sprayed up and down so that the leaves of the tea trees were basically moist. The amount of hormone solution per pot was 50 mL each time, and it was sprayed once every 30 days for a total of three times. The leaf area, chlorophyll content, and photosynthetic rates of the tea tree seedlings were determined 90 days after spraying with the hormone solutions. The second inverted leaves of the tea seedlings were collected to determine the superoxide dismutase activity of leaves, as described in *Section 2.4*.

### 2.7 Statistical analysis

Excel 2020 was used to perform preliminary statistics on raw data. RStudio software (version 4.2.3) was used for graphic production of post-statistical data. The R packages used for the box plots, heat maps, principal component plots, orthogonal partial least squares discrimination analysis (OPLS-DA) models, bubble feature plots, redundancy analysis, and interaction network diagrams were gghalves 0.1.4, pheatmap 1.0.12, ggbiplot 0.55, ropls and mixOmics, ggplot2 3.4.0, vegan 2.6.4, and linkET 0.0.7.1, respectively.

## 3 Results and discussion

### 3.1 Growth indexes and yield analysis of the tea trees

**Figure 1:**
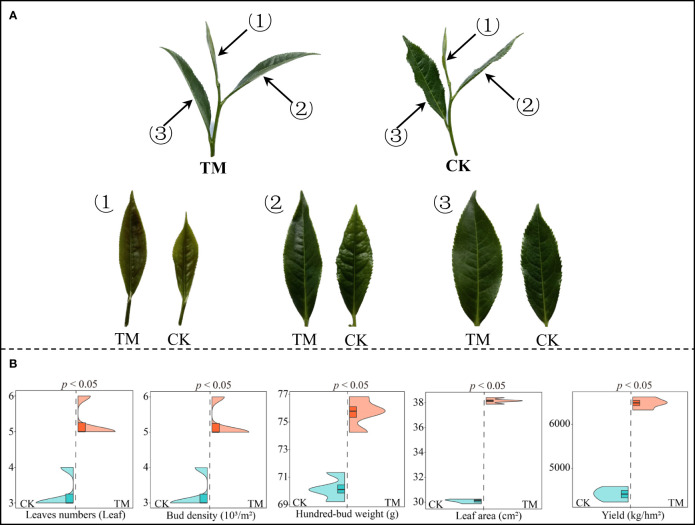
Tea tree growth indexes and yield analysis. *CK*, non-mutagenic Dahongpao tea tree; *TM*, aviation mutagenic Dahongpao tea tree. **(A)** Morphological observation of the inverted first three leaves of CK and TM tea trees. **(B)** Growth indexes and yield analysis of tea trees.

Aviation mutagenesis is highly susceptible to significant changes in the gene and physiological properties of plants, which in turn affect their morphology and growth (Yang et al., 2015). In this study, tea trees after aviation mutagenesis (TM) and tea trees without aviation mutagenesis (CK) were used to analyze the effect of aviation mutagenesis on tree growth. The results showed (**Figure 1A**) that the leaf length, leaf width, and leaf area of the inverted first three leaves of TM tea trees were significantly higher than those of CK trees. In addition, the leaves of TM tea trees had a smoother surface, with the edges serrated more closely and regularly. It can also be observed that the morphology of the tea tree leaves changed significantly after aviation mutagenesis. Further analysis of the growth indexes and yield of tea trees revealed (**Figure 1B**) that the leaf number, bud density, hundred-bud weight, leaf area, and yield of TM tea trees were significantly higher than those of CK trees, which were from 3.25 to 5.25 leaves, from 3.50 to 5.25 × 10^3^/m^2^, from 70.20 to 75.66 g, from 30.09 to 38.16 cm^2^, and from 4,412.50 to 6,482.25 kg/hm^2^, respectively. Moreover, it can be observed that, after aviation mutagenesis, the leaf morphology of the tea trees changed significantly, the growth indexes increased significantly, and the yield increased and showed more superior traits.

### 3.2 Physiological index analysis of tea leaves

**Figure 2:**
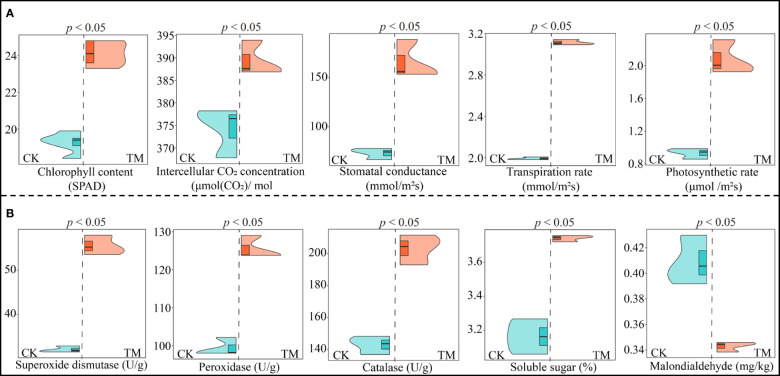
Analysis of the photosynthetic physiology and resistance physiological indexes of tea tree leaves. *CK*, non-mutagenic Dahongpao tea tree; *TM*, aviation mutagenic Dahongpao tea tree. **(A)** Analysis of the photosynthetic physiological indexes of tea tree leaves. **(B)** Analysis of the resistance physiological indexes of tea tree leaves.

Plant growth is closely related to photosynthetic capacity and resistance, and plants with high photosynthetic capacity and resistance more easily adapt to environmental changes and are more conducive to growth (Chang et al., 2022; Wang et al., 2024). Therefore, this study further analyzed the effects of aviation mutagenesis on the photosynthetic physiology and the resistance physiological indexes of the tea trees. Analysis of the photosynthetic physiological indexes of the tea leaves showed that all photosynthetic physiological indexes of TM tea leaves were significantly higher than those of CK leaves, as evidenced by the fact that the chlorophyll content, intercellular CO_2_ concentration, stomatal conductance, transpiration rate, and photosynthetic rate of TM tea leaves were 1.25, 1.04, 1.26, 1.56, and 2.24 times higher than those of CK leaves, respectively (**Figure 2A**). Analysis of the physiological resistance indexes of the tea leaves showed that the superoxide dismutase, peroxidase, and catalase activities and the soluble sugar content of TM tea leaves were significantly higher than those of CK leaves, being 1.74, 1.26, 1.42, and 1.18 times higher than those of CK, respectively (**Figure 2B**). However, the malondialdehyde content of CK was significantly higher than that of TM, being 1.19 times higher. Malondialdehyde has been reported to be associated with plant antioxidant capacity, and its increased accumulation in plants reduces the plant antioxidant capacity, with reduced plant resistance and accelerated senescence (Liu et al., 2015). It can be observed that, after aviation mutagenesis, the photosynthetic capacity and the resistance of the tea trees were enhanced, being more conducive to promoting the growth of tea trees and, thus, increasing the tea yield.

### 3.3 Hormone metabolome analysis of tea tree leaves

**Figure 3:**
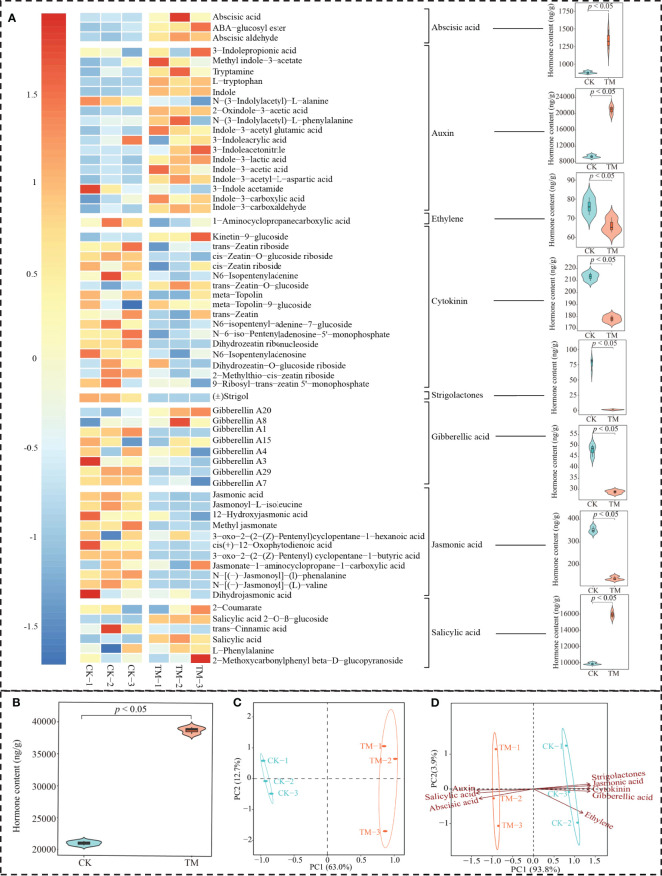
Hormone metabolome analysis of tea tree leaves. *CK*, non-mutagenic Dahongpao tea tree; *TM*, aviation mutagenic Dahongpao tea tree. **(A)** Heat map of 63 hormone contents in TM and CK and their content analysis after categorization. **(B)** Analysis of the total amount of hormones in TM and CK. **(C)** Principal component analysis of the hormone contents in TM and CK. **(D)** Principal component analysis after the categorization of 63 hormones.

Phytohormones are a class of organic substances produced by the plant’s metabolism and are found at low levels in plants. There are many types of phytohormones that play important regulatory roles during the different stages of plant growth and development, especially in the regulation of plant physiology and metabolism, photosynthesis, and resistance (Müller and Munné-Bosch, 2021). Lower levels of phytohormones are highly susceptible to plant photosynthetic capacity and resistance, which in turn affect plant growth (Yuan et al., 2023). In this study, the effect of aviation mutagenesis on the hormone metabolome of tea tree leaves was analyzed. A total of 63 hormones were detected in tea tree leaves, which could be classified into eight groups: abscisic acid, auxin, cytokinin, ethylene, gibberellic acid, jasmonic acid, salicylic acid, and strigolactones (**Figure 3A**). TM contained significantly more abscisic acid, auxin, and salicylic acid than CK, while cytokinin, ethylene, gibberellic acid, jasmonic acid, and strigolactones were significantly lower than those in CK. Further analysis revealed (**Figure 3B**) that the total amount of hormones in TM reached 38.67 mg/g, which was significantly higher than the 21.99 mg/g in CK. The results of the principal component analysis (PCA) of the hormone contents of TM and CK showed that there was a significant difference between the two in terms of hormone type and content and that the two principal components could effectively distinguish between them, with an overall contribution rate of 85.70% (**Figure 3C**).

On this basis, further PCA of the categorized hormones revealed that the hormones significantly associated with TM were abscisic acid, auxin, and salicylic acid, whereas those significantly associated with CK were cytokinin, ethylene, gibberellic acid, jasmonic acid, and strigolactones (**Figure 3D**). Several types of hormones can be observed in the tea tree leaves, and aviation mutagenesis induced significant changes in the total amount of hormones and the contents of the different types of hormones in tea tree leaves.

### 3.4 Screening for characteristic hormones in tea tree leaves

**Figure 4:**
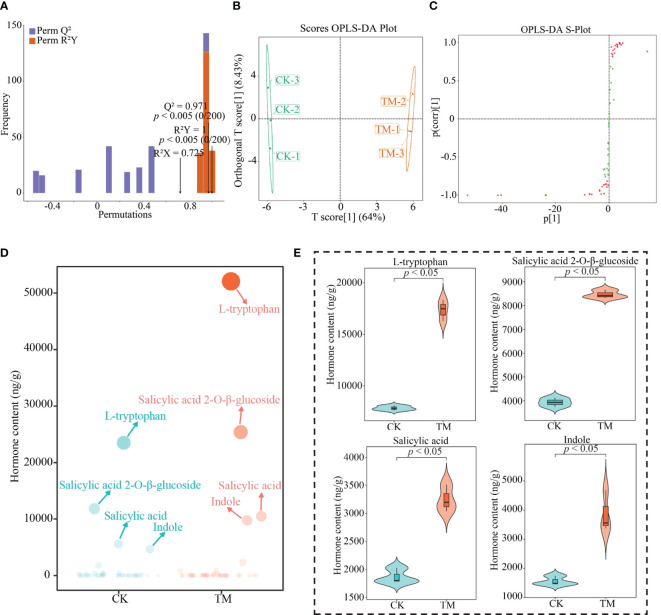
Screening for characteristic hormones in tea tree leaves. *CK*, non-mutagenic Dahongpao tea tree; *TM*, aviation mutagenic Dahongpao tea tree. **(A)** Orthogonal partial least squares discrimination analysis (OPLS-DA) model test plots of CK and TM. **(B)** OPLS-DA model score plots of CK and TM. **(C)** OPLS-DA model S-plot plots of key hormone screening in CK and TM. **(D)** Bubble feature plots of the screening of characteristic hormones. **(E)** Characteristic hormone content analysis.

In order to obtain the characteristic hormones that changed significantly between CK and TM, this study performed further screening using the OPLS-DA model and bubble feature plots. The results showed that the constructed models achieved significant levels of fit (*R* ^2^ *Y*) and predictability (*Q* ^2^) (**Figure 4A**), were able to effectively discriminate between CK and TM (**Figure 4B**), and obtained 39 key hormones for distinguishing between CK and TM (**Figure 4C**). These 39 key hormones were analyzed using bubble feature plots in order to obtain the characteristic hormones with significant differences, which resulted (**Figure 4D**) in four characteristic hormones obtained, namely, l-tryptophan, indole, salicylic acid, and salicylic acid 2-*O*-β-glucoside, which accounted for more than 90% of the total hormones. Further analysis revealed (**Figure 4E**) that all four characteristic hormones were significantly greater in TM than in CK, as evidenced by the l-tryptophan, indole, salicylic acid, and salicylic acid 2-*O*-β-glucoside contents in TM of 17,355.84, 3,493.98, 3,249.54, and 8,463.50 ng/g, with CK only having 7,835.25, 1,562.43, 1,868.71, and 3,935.49 ng/g, respectively. Salicylic acid is commonly found in plants and is typically stored as salicylic acid 2-*O*-β-glucoside, both of which play an important role in the regulation of plant resistance, with an increase in their content conducive to improving plant resistance, in turn improving the plants’ adaptability to the environment and promoting plant growth (Berim and Gang, 2022; Liu et al., 2023). l-tryptophan and indole, which have similar chemical structures, are precursors of plant growth hormone synthesis and play important roles in the regulation of plant growth and development and photosynthesis, and an increase in their contents is conducive to the enhancement of the photosynthetic capacity of plants, promoting plant growth and increasing yield (Bělonožníková et al., 2022; Tivendale and Millar, 2022). It can be observed that, after aviation mutagenesis, the ability of the tea trees to synthesize and accumulate characteristic hormones was enhanced, in which the increase in the contents of l-tryptophan and indole was conducive to the enhancement of the growth and photosynthetic ability of tea trees, while the increase in the contents of salicylic acid and salicylic acid 2-*O*-β-glucoside was conducive to the enhancement of the resistance of tea trees and their adaptability to the environment, in turn promoting their growth.

### 3.5 Interaction analysis

**Figure 5:**
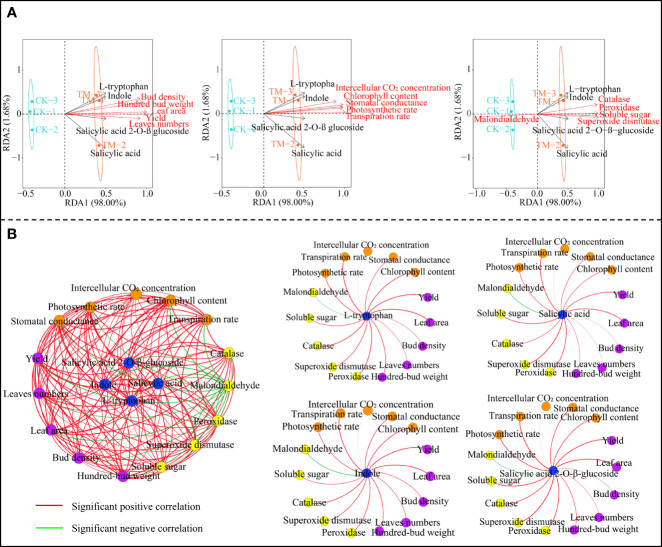
Interaction analysis between the characteristic hormones and the tea tree growth indexes, tea yield, and physiological indexes. *CK*, non-mutagenic Dahongpao tea tree; *TM*, aviation mutagenic Dahongpao tea tree. **(A)** Redundancy analysis of the characteristic hormones with the tea tree growth indexes, tea yield, and physiological indexes. **(B)** Correlation network analysis of the characteristic hormones with the tea tree growth indexes, tea yield, and physiological indexes.

Based on the above analysis, this study further analyzed the interactions between the four characteristic hormones and the growth indexes, tea yield, and physiological indexes of the tea trees. Redundancy analysis showed that the characteristic hormones, the tea tree growth indexes, the tea yield, the photosynthetic physiological indexes, and the resistance physiological indexes (except malondialdehyde) were significantly correlated with TM, while only malondialdehyde was significantly correlated with CK (**Figure 5A**). Correlation network analysis showed that all four characteristic hormones were significantly positively correlated with the tea tree growth indexes, tea yield, and the photosynthetic physiological and resistance physiological indexes, while they were significantly negatively correlated with malondialdehyde (**Figure 5B**). Moreover, it can be observed that the four characteristic hormones obtained in this study were indeed closely related to the growth, photosynthesis, and resistance of tea trees, while aviation mutagenesis improved the accumulation of these characteristic hormones in tea trees, enhanced the photosynthetic capacity, improved the resistance, promoted tree growth, and then improved tea yield.

### 3.6 Effects of the characteristic hormones on the growth and physiology of tea trees

**Figure 6:**
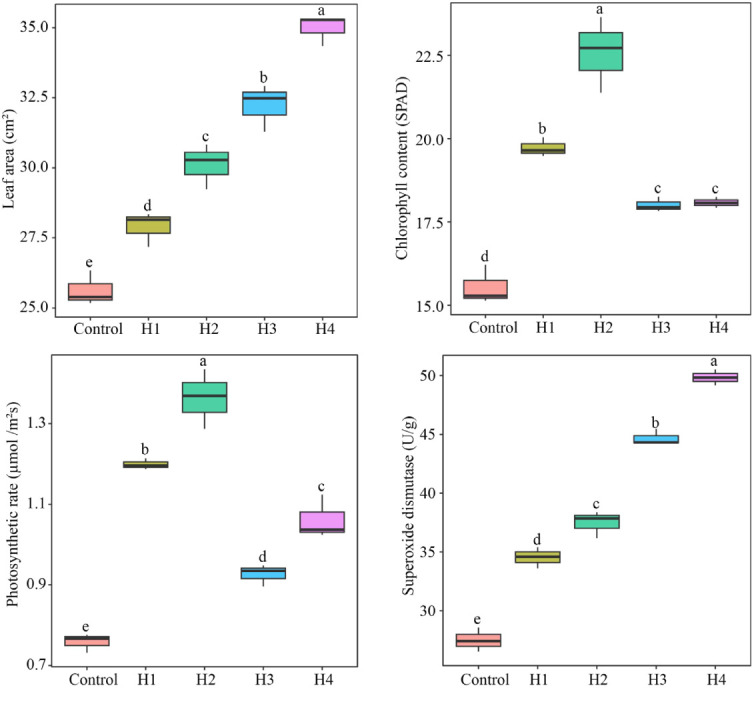
Effects of characteristic hormone spraying treatments on the growth and physiological indexes of tea tree seedlings. *Control*, tea seedlings not sprayed with hormone; *H1*, tea seedlings sprayed with 17.35 g/L l-tryptophan; *H2*, tea tree seedlings sprayed with 3.50 g/L indole; *H3*, tea tree seedlings sprayed with 3.25 g/L salicylic acid; *H4*, tea tree seedlings sprayed with 8.45 g/L salicylic acid 2-*O*-β-glucoside. *Different lowercase letters* indicate differences between samples at the *p* < 0.05 level.

It was found that the characteristic hormones distinguishing TM from CK were l-tryptophan, indole, salicylic acid, and salicylic acid 2-*O*-β-glucoside, and the contents of all four characteristic hormones were significantly higher in TM than in CK. Moreover, the contents of the four characteristic hormones were significantly and positively correlated with the growth and physiological indexes (except malondialdehyde) of tea trees. Therefore, exogenous spraying of these hormones in tea tree seedlings was carried out to set the concentration based on the contents of the four characteristic hormones detected in TM in order to analyze the effects of these hormones on the growth and physiology of tea trees. The results showed that exogenous spraying of all four characteristic hormones significantly increased the leaf area, chlorophyll content, photosynthetic rate, and superoxide dismutase activity of the tea tree leaves (**Figure 6**). This confirms previous conclusions. In addition, this study found that salicylic acid and salicylic acid 2-*O*-β-glucoside were more favorable to increase the leaf area and superoxide dismutase activity of the tea tree leaves, especially salicylic acid 2-*O*-β-glucoside, whereas l-tryptophan and indole were more favorable to increase the chlorophyll content and photosynthetic rate of the tea tree leaves, especially indole. Salicylic acid and salicylic acid 2-*O*-β-glucoside have been reported to have more pronounced effects on plant resistance, and they promote plant growth mainly by regulating plant resistance (Cui et al., 2021; Qiu et al., 2023). l-tryptophan and indole are hormones that belong to the phytogrowth hormone class, and their main role is to promote plant growth, which is more obvious in the increased plant photosynthetic capacity and growth rate (Mir et al., 2020; Xiang et al., 2023). It can be observed that the increase in the contents of the four characteristic hormones in the leaves of tea trees after aviation mutagenesis worked together to improve the tea tree growth rate and resistance, in turn increasing the tea yield.

## 4 Conclusion

In this study, differences in the morphology, growth, physiology, and hormone metabolism of tea trees with aviation mutagenesis (TM) and those without aviation mutagenesis (CK) were analyzed. The results showed that tea trees after aviation mutagenesis had obvious changes in leaf morphology, had enhanced photosynthetic capacity and resistance, and had significantly improved growth indexes and yield. Hormone metabolome analysis revealed that the characteristic hormones distinguishing TM from CK were l-tryptophan, indole, salicylic acid, and salicylic acid 2-*O*-β-glucoside, with the contents of all four characteristic hormones being significantly higher in TM than in CK. Interaction analysis showed that these four characteristic hormones were significantly positively correlated with the growth indexes, tea yield, and the photosynthetic and resistance physiological indexes of the tea trees, except malondialdehyde. After exogenous spraying of tea tree seedlings with these characteristic hormones, it was found that all four characteristic hormones significantly increased the leaf area, chlorophyll content, photosynthetic rate, and superoxide dismutase activity of the tea tree leaves, of which salicylic acid and salicylic acid 2-*O*-β-glucoside were more favorable to increase the leaf area and superoxide dismutase activity, whereas l-tryptophan and indole were more favorable to increase the chlorophyll content and photosynthetic rate of tea leaves. It can be observed that aviation mutagenesis improved the accumulation of these characteristic hormones in tea trees, enhanced the photosynthetic capacity, improved the resistance of tea trees and promoted their growth, and consequently improved the tea yield. This study analyzed the effects of aviation mutagenesis on the morphology, growth, and physiological properties of tea trees and their hormone regulation mechanism, providing a reference for the development of aviation mutagenesis of tea trees and laying a foundation for the application and promotion of aviation mutagenesis in tea trees. However, changes in the soil ecosystem during the planting process have certain impacts on the growth of tea trees. Is there a difference in the rhizosphere soil microorganisms and nutrients between aviation mutagenic and non-aviation mutagenic tea trees? Does this difference have an effect on the growth of tea tree? Further exploration is needed.

## Data availability statement

The datasets presented in this study can be found in online repositories. The names of the repository/repositories and accession number(s) can be found in the article/**Supplementary Material**.

## Author contributions

MJ: Writing – original draft, Visualization, Methodology, Conceptualization. YC: Writing – original draft, Visualization, Methodology, Conceptualization. QZ: Writing – original draft, Formal analysis. YW: Writing – original draft, Formal analysis. ML: Writing – original draft, Formal analysis. XP: Writing – original draft, Formal analysis. LH: Writing – original draft, Methodology, Investigation. SL: Writing – original draft, Methodology, Investigation. XJ: Writing – original draft, Methodology, Investigation. JY: Writing – review & editing, Writing – original draft, Supervision, Resources, Project administration, Methodology, Funding acquisition. HW: Writing – review & editing, Writing – original draft, Supervision, Resources, Project administration, Methodology, Funding acquisition.
