# Supplementary material for: Changes in the growth and physiological property of tea tree after aviation mutagenesis and screening and functional verification of its characteristic hormones
Source: Front Plant Sci. 2024 Jul 24;15:1402451. doi: 10.3389/fpls.2024.1402451 (PMC11303228; doi:10.3389/fpls.2024.1402451)
Supplement: Supplementary file 1 [file DataSheet_1.pdf]

Supplementary data

Table S1 Standard curves for different hormones

Index	Class	Equation	r	LLOQ	ULOQ
ABA-glucosyl ester	ABA	y = 0.43095 x + 0.00688	0.9996	5	500
Abscisic acid	ABA	y = 0.10105 x + 2.48791e-4	0.99866	0.1	500
Abscisic aldehyde	ABA	y = 0.01991 x - 9.77245e-5	0.99864	5	500
L-tryptophan	Auxin	y = 0.00661 x + 0.00601	0.99922	2	10000
Tryptamine	Auxin	y = 0.12885 x + 0.01927	0.99918	0.1	500
2-oxindole-3-acetic acid	Auxin	y = 0.01123 x + 5.43799e-4	0.99953	1	500
Indole-3-acetyl-L-aspartic acid	Auxin	y = 0.01829 x + 2.16551e-4	0.99917	0.1	500
3-Indole acetamide	Auxin	y = 0.48867 x + 0.00843	0.99658	0.1	500
Indole-3-acetyl glutamic acid	Auxin	y = 0.15697 x - 0.00358	0.99641	0.1	500
Indole-3-lactic acid	Auxin	y = 0.01702 x + 0.00171	0.99904	2	500
N-(3-Indolylacetyl)-L-alanine	Auxin	y = 0.27002 x + 0.00629	0.99966	0.1	500
Indole-3-carboxylic acid	Auxin	y = 0.00990 x + 5.62028e-4	0.99972	0.5	500
Indole-3-carboxaldehyde	Auxin	y = 0.04384 x + 0.03809	0.99589	0.1	500
Indole-3-acetic acid	Auxin	y = 0.01421 x + 5.84948e-4	0.99987	0.2	500
3-Indoleacrylic acid	Auxin	y = 0.01364 x + 5.96593e-4	0.99901	0.2	500
3-Indolepropionic acid	Auxin	y = 0.03954 x + 0.00101	0.99946	0.1	500
3-Indoleacetonitrile	Auxin	y = 0.03344 x + 0.00346	0.99816	0.5	500
N-(3-Indolylacetyl)-L-phenylalanine	Auxin	y = 0.59739 x + 0.00818	0.9981	0.5	500
Methyl indole-3-acetate	Auxin	y = 0.07751 x + 0.00129	0.99149	0.2	500
Indole	Auxin	y = 0.00480 x + 0.00318	0.99917	0.5	500

9-Ribosyl-trans-zeatin 5'-monophosphate	CK	y = 0.02697 x + 4.37114e-5	0.9922	2	500
trans-Zeatin-O-glucoside	CK	y = 0.10189 x - 1.35886e-4	0.99948	0.2	500
trans-Zeatin	CK	y = 0.17074 x + 0.00312	0.99961	0.1	500
Dihydrozeatin-O-glucoside riboside	CK	y = 0.38607 x + 0.00347	0.99982	0.1	500
cis-Zeatin-O-glucoside riboside	CK	y = 0.02371 x + 1.76233e-4	0.99665	0.1	500
trans-Zeatin riboside	CK	y = 0.15341 x + 0.00186	0.9957	0.1	500
Dihydrozeatin ribonucleoside	CK	y = 0.08187 x + 0.00125	0.99038	0.1	500
cis-Zeatin riboside	CK	y = 0.10159 x + 7.57348e-4	0.99045	0.1	500
N6-Isopentenyl-adenine-7-glucoside	CK	y = 0.40017 x + 0.00404	0.9921	0.1	500
meta-Topolin-9-glucoside	CK	y = 0.14867 x + 0.00223	0.99071	0.1	500
meta-Topolin	CK	y = 0.11331 x + 8.87902e-4	0.9962	2	500
Kinetin-9-glucoside	CK	y = 0.23783 x + 0.00367	0.99198	0.1	500
N-6-iso-pentenyladenosine-5'-monophosphate	CK	y = 0.10998 x - 6.20323e-4	0.9974	1	500
N6-isopentenyladenine	CK	y = 0.07011 x + 0.00776	0.99672	0.1	500
N6-isopentenyladenosine	CK	y = 0.08602 x + 0.00118	0.99055	0.1	500
2-Methylthio-cis-zeatin riboside	CK	y = 0.13056 x + 0.00331	0.99205	0.1	500
1-Aminocyclopropanecarboxylic acid	ETH	y = 17969.90028 x + 26108.08314	0.99588	1	500
Gibberellin A8	GA	y = 0.09780 x + 0.00351	0.99527	2	500
Gibberellin A29	GA	y = 0.00418 x + 0.00307	0.99641	2	500
Gibberellin A3	GA	y = 0.06340 x + 0.00488	0.99737	1	500
Gibberellin A1	GA	y = 0.02161 x + 0.01473	0.99369	2	500
Gibberellin A20	GA	y = 0.01764 x + 0.00254	0.99403	2	500
Gibberellin A7	GA	y = 0.27391 x + 0.02263	0.99847	1	500
Gibberellin A4	GA	y = 0.02691 x + 0.00696	0.99135	2	500

Gibberellin A15	GA	y = 0.02213 x + 0.00111	0.99481	2	500
12-Hydroxyjasmonic acid	JA	y = 0.03420 x + 0.04417	0.99756	5	500
Jasmonate-1-aminocyclopropane-1-carboxylic acid	JA	y = 0.04594 x + 0.00170	0.99036	0.5	500
Jasmonic acid	JA	y = 0.02661 x + 0.00142	0.99666	0.2	500
N-[(-)-Jasmonoyl]-(L)-valine	JA	y = 0.28739 x + 0.00378	0.99701	0.1	500
Dihydrojasmonic acid	JA	y = 0.03599 x + 0.00594	0.99925	1	10000
Jasmonoyl-L-isoleucine	JA	y = 0.05999 x + 0.00124	0.99223	0.1	500
N-[(-)-Jasmonoyl]-(l)-phenalanine	JA	y = 0.14452 x + 0.00294	0.99836	0.1	500
3-oxo-2-(2-(Z)-Pentenyl) cyclopentane-1-butyric acid	JA	y = 0.01425 x + 0.01694	0.99307	2	500
Methyl jasmonate	JA	y = 0.45138 x + 0.01370	0.99993	0.2	500
3-oxo-2-(2-(Z)-Pentenyl)cyclopentane-1-hexanoic acid	JA	y = 0.02245 x + 0.01610	0.99658	5	500
cis(+)-12-Oxophytodienoic acid	JA	y = 0.32758 x + 0.01221	0.99835	0.1	500
L-Phenylalanine	SA	y = 718.20311 x + 20048.99614	0.99186	1	15000
Salicylic acid 2-O-β-glucoside	SA	y = 0.03091 x + 0.00919	0.99259	1	10000
2-Methoxycarbonylphenyl beta-D-glucopyranoside	SA	y = 3.85630e4 x + 23844.09792	0.99949	20	500
2-Coumarate	SA	y = 6.37981e4 x + 16791.33010	0.9986	10	500
Salicylic acid	SA	y = 0.10368 x + 0.02072	0.99967	0.5	500
trans-Cinnamic acid	SA	y = 235.67156 x + 920.82651	0.99904	200	10000
（±）Strigol	SL	y = 579.65709 x + 207.02849	0.99695	20	10000

Note: Index: Hormone name; Class: Classification of hormones; Equation: Linear equation; r: Coefficient of Correlation; LLOQ: Lower limit of quantitation (ng/mL); ULOQ: Higher limit of quantitation (ng/mL).
